# Development and validation of proton track-structure model applicable to arbitrary materials

**DOI:** 10.1038/s41598-021-01822-1

**Published:** 2021-12-21

**Authors:** Tatsuhiko Ogawa, Yuho Hirata, Yusuke Matsuya, Takeshi Kai

**Affiliations:** Japan Atomic Energy Agency, Research Group for Radiation Transport Analysis, Tokai, Ibaraki 319-1195 Japan

**Keywords:** Materials science, Nanoscience and technology

## Abstract

A novel transport algorithm performing proton track-structure calculations in arbitrary materials was developed. Unlike conventional algorithms, which are based on the dielectric function of the target material, our algorithm uses a total stopping power formula and single-differential cross sections of secondary electron production. The former was used to simulate energy dissipation of incident protons and the latter was used to consider secondary electron production. In this algorithm, the incident proton was transmitted freely in matter until the proton produced a secondary electron. The corresponding ionising energy loss was calculated as the sum of the ionisation energy and the kinetic energy of the secondary electron whereas the non-ionising energy loss was obtained by subtracting the ionising energy loss from the total stopping power. The most remarkable attribute of this model is its applicability to arbitrary materials, i.e. the model utilises the total stopping power and the single-differential cross sections for secondary electron production rather than the material-specific dielectric functions. Benchmarking of the stopping range, radial dose distribution, secondary electron energy spectra in liquid water, and lineal energy in tissue-equivalent gas, against the experimental data taken from literature agreed well. This indicated the accuracy of the present model even for materials other than liquid water. Regarding microscopic energy deposition, this model will be a robust tool for analysing the irradiation effects of cells, semiconductors and detectors.

## Introduction

As tools for predicting the macroscopic behaviour of radiation, general-purpose radiation transport codes are used in various domains of radiation application^1–10^. The microscopic resolution of these codes has recently been improved using track-structure calculations^11^, where charged particle transport is calculated with explicit consideration for every projectile-target reaction. Conventionally, the simulation of charged particles is based on the condensed history method. In this method, the charged particles are transported in matter with energy depositing along the particle track until the particle interacts with a nucleus or reaches material boundary. The microscopic details of energy deposition are disregarded in this method. In contrast, track-structure calculations use cross sections to explicitly simulate each energy deposition reaction attributed to atomic interaction. These calculations can therefore achieve sub-micron spatial resolution and sub-keV energy resolution. Owing to these attributes, track-structure models have been developed worldwide^12–30^ and have been applied in various fields, such as radiation biology^31–33^, radiation medicine^34–36^, chemical impact of radiation^37,38^, detector physics^39–41^ and semiconductor technologies^17,42^.

Cross section data on atomic reactions between the primary particle and target atoms, which are based on the dielectric function of the target material^43^, is an essential ingredient of track-structure calculations. However, except for liquid water^44^, the dielectric function has not been measured over a wide frequency range. In addition, reliable theoretical methods which can globally predict the dielectric function of arbitrary materials have yet to be reported. Conventional track-structure models are almost exclusively^20^ applicable to liquid water. Moreover, demand has risen in various fields (e.g. DNA for radiation biology, luminescence materials, ionising gases for detector physics and Si for semiconductor applications) for track-structure calculation models applicable to materials other than liquid water. To expand the applicability of these models to new materials, an innovative approach that requires no dielectric function data is necessary.

A model capable of simulating the track-structure in arbitrary materials of commonly used charged particles is required. Electrons are one of the most important particles and a study has suggested that an electron track-structure algorithm developed for liquid water can be applied to other materials by scaling the cross sections by the electron density^10^. Conventional models are fairly effective in performing electron track-structure calculations for materials other than liquid water. The proton is the second-most commonly used charged particle and secondary electron production by protons is material dependent, as indicated by Rudd^45^. Therefore, the development of a track-structure calculation model for protons applicable in arbitrary materials is highly desirable.

In this study, a track-structure calculation algorithm for protons is developed by combining a systematic formula of the total stopping power with that of the secondary electron energy distribution. The stopping range, secondary electron energy distribution, and radial dose distribution in liquid water calculated by the developed model (Ion Track Structure model for Arbitrary Radiation and Targets, referred to as ITSART hereafter) were compared with corresponding measurement data taken from the literature. This comparison revealed that the developed model can perform accurate track-structure calculations without using dielectric functions. ITSART was then used to calculate the microscopic energy distribution in a tissue-equivalent gas (a gas composed of 54.7% ${{\hbox{C}}}_{3}{{\hbox{H}}}_{8}$, 39.7% ${{\hbox{CO}}}_{2}$ and 5.6% ${\hbox{N}}_{2}$ in molar ratio, hereafter TEG). The distribution was compared with the measurement data. The good agreement realised indicates the validity of ITSART for materials other than liquid water.

## Method

### Theory of our track-structure calculation model

The interaction of incident protons and the target material consists of an ionising interaction and a non-ionising interaction. The latter involves excitation (e.g. phonon, vibration and rotation), and elastic scattering, whereas the former involves ionisation. In our model, ionisation was simulated on the basis of a cross section systematic formula proposed by Rudd^45^. The ionisation cross sections of electrons in each shell of the target molecules were calculated using the Rudd’s formula. By combining the calculated cross sections with a random number, 4-dimensional coordinates (x, y, z, time) and the source shell of the electron were determined. Rudd’s formula provides the ionisation cross section and the secondary electron energy distribution; hence, the ejected secondary electron energy was also sampled. Furthermore, the emission angle of the electron and the momentum of the projectile after the reaction were determined by energy and momentum conservation. Usually, the conservation of momentum and energy was satisfied between the projectile and the secondary electron but conservation of longitudinal momentum was unattainable when the electron was ejected at a very forward angle. In this case, the remainder of momentum (typically on the order of 0.1 MeV/c) was given to the target atom. The average ionisation energy loss of the incident particle was also calculated using the cross section of each target electron shell and the secondary electron energy distribution (the calculation procedure is explained in a subsequent section). All the secondary electrons were transported further by the electron track-structure model of PHITS^19,46^ (hereafter referred to as ETS-mode). Even for targets other than liquid water, this model is applicable owing to the scaling of cross sections by the electron density^10^.

**Figure 1:**
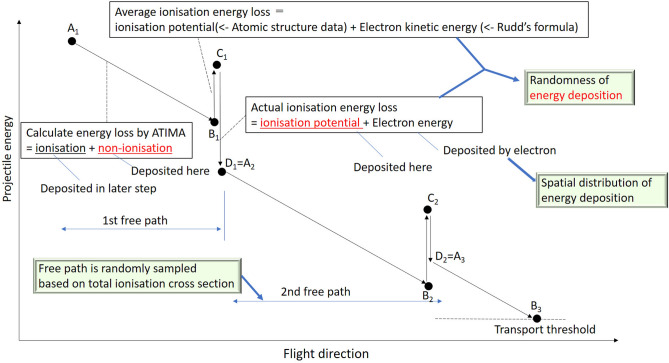
Diagram of showing the restricted energy loss calculation. In each free path, the projectile energy decreases based on the stopping power and secondary electron production cross sections.

ITSART uses the secondary electron production cross section and the total stopping power of the projectiles, which comprises the non-ionisation energy loss and ionising energy loss, to calculate the energy loss of the projectile. To avoid double counting of the ionising energy loss, the non-ionisation energy loss was calculated by subtracting the average ionisation energy loss (calculated above) from the total stopping power. This energy dissipation balance calculation referred to as the restricted stopping power calculation (see Fig. 1) worked as a bypass for simulating ion transport without knowledge of the non-ionising reaction cross-sections.

A free flight path length *dL* of proton with kinetic energy $E_{\mathrm{p}}$ was determined by ionisation cross section,

1 $$\begin{aligned} dL = \frac{-{\mathrm{Log}}(r)}{ \Sigma _{\mathrm{ion}}\big (E_{\mathrm{p}}\big ) }, \end{aligned}$$

where *r* is a uniform random number from 0 to 1 and $\Sigma _{\mathrm{ion}}$ is the macroscopic ionising reaction cross section. Given that $S_{\mathrm{tot}}(E)$ is the total stopping power of proton with kinetic energy *E*, the total energy loss in this path $dE_{\mathrm{tot}}$ with length dL is given as;

2 $$\begin{aligned} dE_{\mathrm{tot}} = S_{\mathrm{tot}}\big (E_{\mathrm{p}}\big ) dL. \end{aligned}$$

The right hand side of Eq. (2) consists of two components,

3 $$\begin{aligned} dE_{\mathrm{tot}} = \overline{\varepsilon _{\mathrm{non}}} \Sigma _{\mathrm{non}}\big (E_{\mathrm{p}}\big ) dL + \overline{\varepsilon _{\mathrm{ion}}} \Sigma _{\mathrm{ion}}\big (E_{\mathrm{p}}\big ) dL, \end{aligned}$$

where $\overline{\varepsilon _{\mathrm{non}}}$ is the mean energy loss induced by non-ionising reactions, $\Sigma _{\mathrm{non}}$ is the macroscopic non-ionising reaction cross section, and $\overline{\varepsilon _{\mathrm{ion}}}$ is the mean energy loss resulting from ionising reactions. Here $\overline{\varepsilon _{\mathrm{non}}}$ and $\Sigma _{\mathrm{non}}$ comprise non-ionising reaction channels and are expressed as follows;

4 $$\begin{aligned} \overline{\varepsilon _{\mathrm{non}}} \Sigma _{\mathrm{non}}\big (E_{\mathrm{p}}\big ) dL = n \sum _{j} \int _{0}^{\infty } E \sigma _{\mathrm{non},j}\big (E,E_{\mathrm{p}}\big ) dE dL, \end{aligned}$$

where *n* is the atomic density and $\sigma _{{\mathrm{non}},j}(E)$ is the microscopic cross section of the *j*-th non-ionising reaction channel associated with loss of kinetic energy *E*.

In contrast, $\overline{\varepsilon _{\mathrm{ion}}}$ and $\Sigma _{\mathrm{ion}}$ are broken down to contributions from electron shells and can be expressed as follows;

5 $$\begin{aligned} \overline{\varepsilon _{\mathrm{ion}}} \Sigma _{\mathrm{ion}}\big (E_{\mathrm{p}}\big ) dL = n \sum _{i} \int _{0}^{\infty } (\varepsilon _{i} + E ) \sigma _{{\mathrm{ion}},i}\big (E,E_{\mathrm{p}}\big ) dE dL, \end{aligned}$$

where $\varepsilon _{i}$ is the ionisation energy of the *i*-th electron shell, and $\sigma _{{\mathrm{ion}},i}(E)$ is the microscopic cross section of electrons in the *i*-th electron shell that produces a secondary electron of kinetic energy *E* calculated from Rudd’s formula.

The energy deposited directly by incident protons (i.e. not by secondary electrons) along the trajectory was the sum of the non-ionisation energy loss and ionisation potential, underlined in Fig. 1. From Eqs. (3)–(5), this quantity is expressed as follows;

6 $$\begin{aligned} dE_{\mathrm{tot}} - n \sum _{i} \int _{0}^{\infty } E \sigma _{{\mathrm{ion}},i}\left( E,E_{\mathrm{p}}\right) dE dL, \end{aligned}$$

where $dE_{\mathrm{tot}}$ was calculated from ATIMA and $\sigma _{{\mathrm{ion}},i}(E,(E_{\mathrm{p}}))$ was calculated from Rudd’s formula. The projectile kinetic energy at C$_{1}$ in Fig. 1 was calculated as follows;

7 $$\begin{aligned} E_{\mathrm{C1}} = E_{\mathrm{A1}} - dE_{\mathrm{tot}} + n \sum _{i} \int _{0}^{\infty } E \sigma _{{\mathrm{ion}},i}\left( E,E_{\mathrm{p}}\right) dE dL + n \sum _{i} \varepsilon _{i} \int _{0}^{\infty } \sigma _{{\mathrm{ion}},i}\left( E,E_{\mathrm{p}}\right) dE dL. \end{aligned}$$

where $E_{\mathrm{A1}}$ is the projectile kinetic energy at A$_{1}$. Since the ionisation potential $\varepsilon _{i}$ was taken from literature, this value can be calculated using the above-mentioned formulae and database.

The energy directly deposited by primary protons does not include the kinetic energy of secondary electrons [as expressed in Eq. (6)], but the kinetic energy was later deposited by the electrons. The reduction in the primary ion energy before secondary electron production [see Eq. (7)] represents the non-ionising energy loss, which neglects the energy loss induced by this production. However, the proton loses its energy in a stochastic manner, owing to the subsequent secondary electron production (as depicted in Fig. 1 as steps from C$_{1}$ to D$_{1}$ and from C$_{2}$ to D$_{2}$). Given that the secondary electron was ejected from *I*-th electron shell with kinetic energy $E'$,

8 $$\begin{aligned} E_{\mathrm{D1}} = E_{\mathrm{C1}} - E' - \varepsilon _{I} . \end{aligned}$$

Eventually, the energy loss averaged along the projectile track agrees with the energy dissipation calculated by ATIMA. Calculations of the non-ionising energy loss in this manner correspond to non-explicit treatment of these loss events. This shortcoming of ITSART had no effects on the simulation of relevant quantities because these events occurred only along the particle trajectory as illustrated in the subsequent section.

Occasionally, the energy at point ${{\hbox{C}}}_{1}$ exceeded that at point ${{\hbox{A}}}_{1}$ because the energy dissipation associated with continuous slowing down and the average ionisation energy loss were calculated from independent formulae, ATIMA and Rudd’s formula, respectively. Nevertheless, regardless of the free path length, the energy at the end of a free path was always lower than that at the beginning, owing to the large energy reduction induced by the ionisation potential.

The ionisation potential data of elements and some compounds (${\hbox{NH}}_{2}$, ${\hbox{CH}}_{3}$, ${\hbox{C}}_{2}{{\hbox{H}}}_{2}$, ${\hbox{C}}_{2}{{\hbox{H}}}_{6}$, ${\hbox{C}}_{6}{{\hbox{H}}}_{6}$, ${\hbox{NH}}_{3}$, ${\hbox{CH}}_{4}$, ${\hbox{C}}_{2}{{\hbox{H}}}_{4}$, ${{\hbox{H}}}_{2}{\hbox{O}}$, ${\hbox{CO}}_{2}$, ${\hbox{SF}}_{6}$, (${\hbox{CH}}_{3}$)$_{2}{\hbox{NH}}$ and ${\hbox{TeF}}_{6}$) was taken from literature^45,47^. Ionisation potentials of the constituent elements were used if the chemical form of the target compound was unspecified or the ionisation potential of the compound was unavailable. As explained later, the cross sections depends mainly on the electrons of the outermost shell. Therefore the binding energy of valence electrons is important for accurate prediction of molecular cross sections and substitutive use of element’s ionisation potential may cause overestimation of cross sections. The parameters of Rudd’s formula (${\hbox{A}}_{1}$, ${\hbox{A}}_{2}$, ${\hbox{B}}_{1}$, ${\hbox{B}}_{2}$, ${\hbox{C}}_{1}$, ${\hbox{C}}_{2}$, ${\hbox{D}}_{1}$, ${\hbox{D}}_{2}$ and ${\hbox{E}}_{1}$ in Table 1 of the Rudd’s publication^45^, hereafter referred to as Rudd’s parameters) were given for the outermost shells of He, Ne, Ar, Kr, ${{\hbox{H}}}_{2}$, ${\hbox{O}}_{2}$, ${{\hbox{H}}}_{2}{\hbox{O}}$, ${\hbox{CO}}_{2}$ and ${\hbox{CH}}_{4}$ and for the inner shells^48^. The total and single-differential secondary electron production cross sections of Fe, for example, were calculated in this manner. For this calculation, the cross sections of the electrons in the K, M and L shells were calculated by using the ionisation potential of the respective K, L and M shell electrons of Fe and Rudd’s parameters for inner shell electrons. In addition, the cross section of two electrons in the N shell was calculated by using the ionisation potential of N shell electrons occurring in Fe and Rudd’s parameters of Kr with an outermost occupied N shell. The cross sections calculated by Rudd’s formula are inversely proportional to the cubic of ionisation potential; hence the contribution of the inner shells to the cross section was substantially smaller than that of the outermost shell. Therefore, in this case, the cross section of Fe depends mainly on the N-shell electrons.

**Figure 2:**
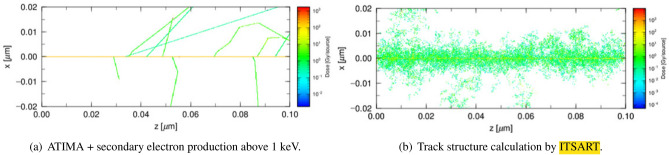
Energy deposition of 1 MeV proton in Si for 100 histories calculated by ITSART and those calculated by conventional continuous slowing-down model.

Although our model neglects coherent scattering by crystalline materials, and the effect of molecular bonds with ionisation potential or Rudd’s parameters are unknown, this approach is more robust than the conventional models. Figure 2 shows a typical proton track-structure calculation performed by our model and conventional transport calculation based on the continuous slowing-down model for 100 histories.

### Benchmarking calculation

For benchmarking, ITSART was implemented in the general-purpose radiation transport code PHITS (Particle and Heavy Ion Transport code System). The transport of electrons, positrons and photons, as well as the scoring of particle track and energy deposition were performed using the corresponding functions of PHITS. Further details of the calculation setup are presented below.

(a) Secondary electron production single-differential cross section

A liquid water or He target of (density: ${10}^{24}$ atoms/cm$^{3}$ and thickness: 10 nm) was exposed to a projectile pencil beam. In the target, the electrons recoiled directly by protons were scored at the time of reaction and those scattered by other electrons were disregarded. The results were averaged over $10^{4}$–$10^{5}$ primary particle histories.

(b) Stopping range

A proton beam was directed toward a 500-cm-thick target of liquid water. The longitudinal coordinate of the protons was scored when the protons reached a transport threshold energy of 1 eV. In other words, the projected range (rather than the total path length) was scored. The coordinate was characterised by a finite distribution, which represents longitudinal straggling; hence, the mean coordinate was calculated. The results were averaged over $10^{2}$–$10^{3}$ primary particle histories.

(c) Radial dose distribution

A cylindrical liquid water target with density of 1 g/cm$^{3}$ was exposed to a proton beam directed perpendicular to the centre of the top surface. The range of secondary electrons was unknown a-priori and the energy deposition depth profile was unimportant; hence, the cylinder (radius: 100 cm and thickness: 100 nm) was fabricated from completely reflective bottom and top surfaces. The radial dose distribution in infinite cylinder was scored by scoring the energy deposition inside this thin cylinder as a function of the radius. To focus on the energy deposition by the projectile at the incident energy, the projectiles were cut off when they lost 0.1% of kinetic energy. Secondary electrons and their electron cascade were transported down to 1 eV.

(d) Lineal energy distribution

The lineal energy (one of microdosimetric quantities^49^, which characterises the energy deposition by the radiation) distributions in spheres of diameter *R* was calculated. For this calculation, a cubic target with side $179\times R$ was filled with spheres (each of diameter *R*) composed of liquid water or TEG with density of 1 g/cm$^{3}$. The target was fabricated with completely reflective surfaces to prevent electron escape from the target and properly calculate the lineal energy in the spheres close to the target surfaces. The projectile protons were started from one of the cubic target surfaces with a random incidence angle (from 0 to 30 degrees) with respect to the target surface. The protons were transported inside the target via the production of secondary electrons until they were cut off losing 0.5% of kinetic energy. For every projectile, the energy deposition in each sphere was scored and divided by the mean chord length ($R \times$ 2/3) in order to obtain the lineal energy.

**Figure 3:**
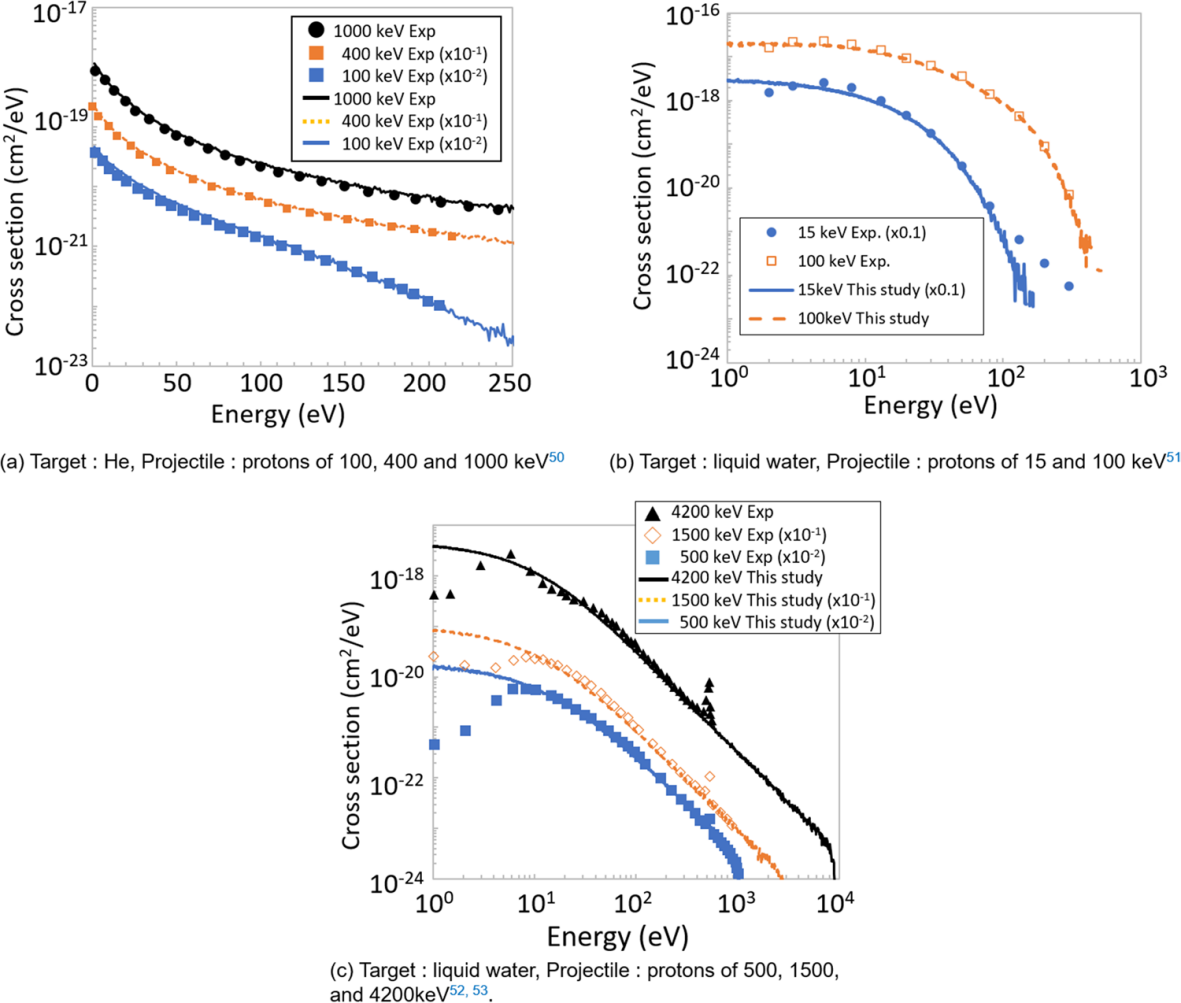
Measured and calculated single-differential secondary electron production cross section.

## Results and discussion

To verify the performance of ITSART, the secondary electron production single-differential cross section, stopping range, radial dose distribution and lineal energy distribution were calculated. Figure 3a compares single-differential He(p,p’e$^{-}$) cross sections for protons with energy of 100, 400 and 1000 keV^50^. These examples show that secondary electrons produced by incident protons are accurately reproduced by our model. The calculation for noble gases should be particularly accurate because these calculations involve no molecular or crystalline structures, which are neglected by our model. Figure 3b and c compare single-differential ${{\hbox{H}}}_{2}{{\hbox{O}}}({\hbox{p,e}}^{-}$) cross sections for protons of 15, 100, 500, 1500, and 4200 keV. As discussed in the literature^51^, the dip of the measured spectra below 10 eV in Fig. 3c is attributed to the deterioration of detection efficiency, therefore this disagreement is meaningless. The high energy tail above 200 eV of the energy spectrum for 15 keV protons in Fig. 3a is an artefact attributed to detector background noise as explained by the authors^52^. Our calculation is reasonable because the authors even state that the energy spectrum should fall exponentially as a function of energy. The peak just above 500 eV represents Auger electrons produced by K-shell ionisation. The reproduction of these electrons requires molecule-specific Auger electron energy and strength and is therefore beyond the scope of this model. Aside from above peaks and artefacts, the uncertainty of experimental data is 11%. The magnitude and slope of the cross sections calculated by our model concur with the experimental data.

**Figure 4:**
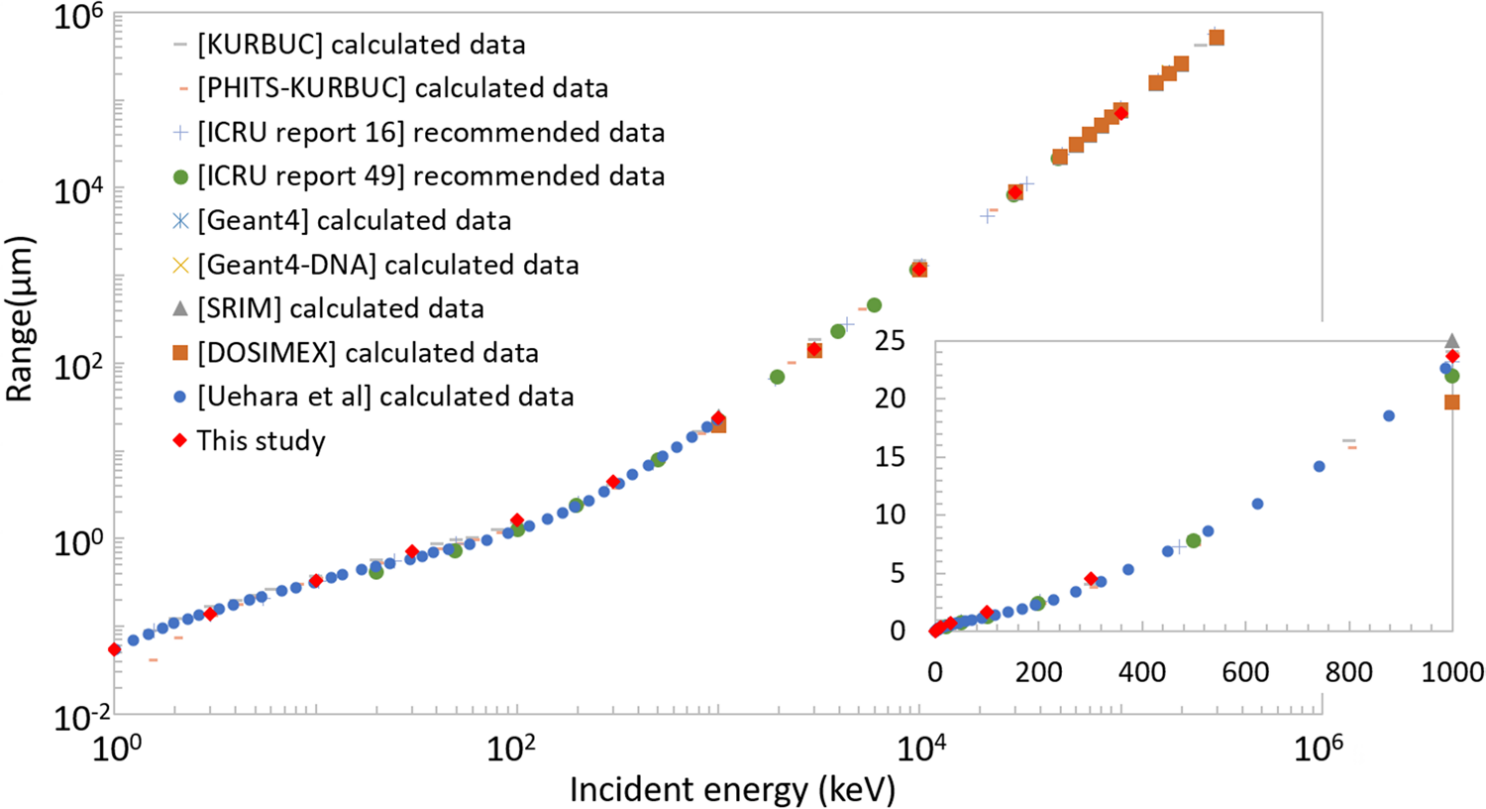
Measured and calculated stopping range of protons in liquid water^6,53–61^. The inset is linear-linear plot in the energy range from 0 to 1000 keV.

Figure 4 shows the range of protons in liquid water^6,53,54,56–61^. Although the stopping power calculated by ATIMA was modulated by the restricted stopping power algorithm, the calculated range concurs with literature data. The modulation of the stopping power by the algorithm occurs in every electron emission; hence, the modulation effect accumulates as the proton travels in the material. Therefore, the good agreement realised for high-energy protons indicates that the modulation cancels out in the macroscopic scale.

**Figure 5:**
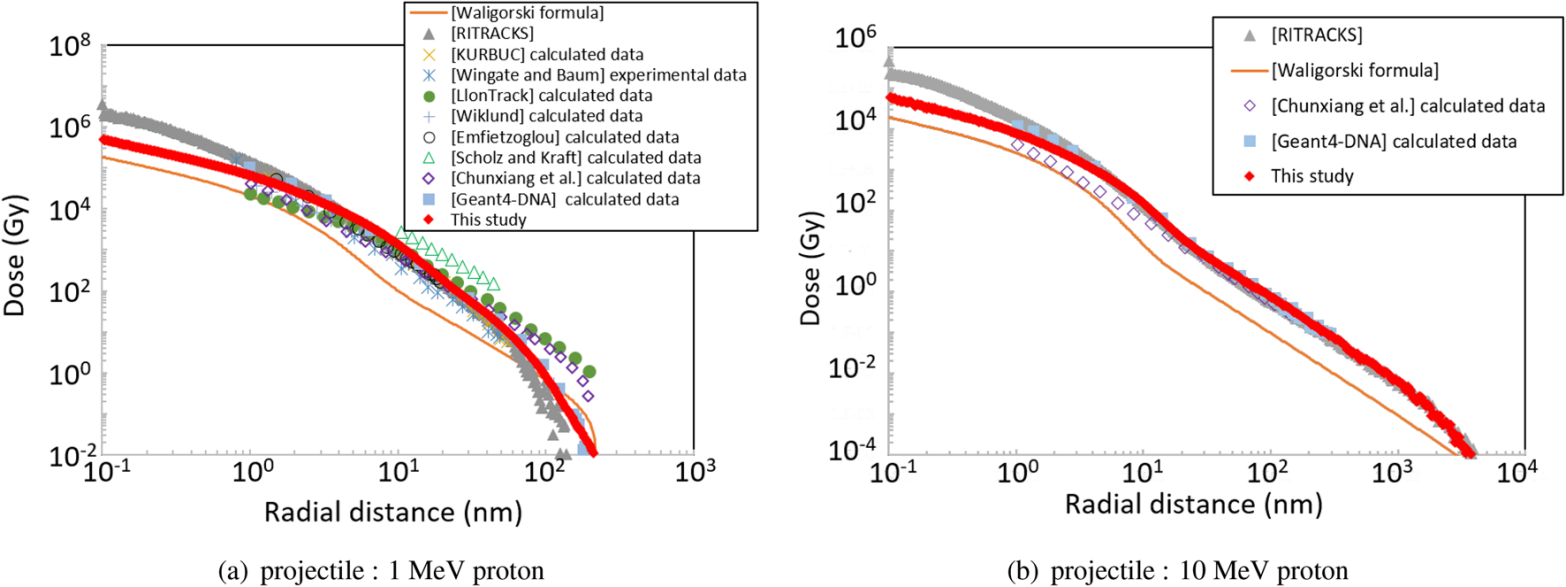
Measured and calculated radial distribution of energy deposited by 1 and 10 MeV protons in liquid water^12,18,55,62–68,70^.

Figure 5a shows the radial distribution of energy deposited by a 1 MeV proton beam. RITRACKS Ver. 3.1, released in 2016, was used to simulate the energy deposited in a water target with thickness 100 nm bombarded by each projectile. To score energy deposition with consideration for electronic equilibrium despite the finite target thickness, RITRACKS simulates secondary electrons until they are thermalised. The calculation by ITSART supplemented by ETS-mode, which simulates the transport of produced secondary electrons to outer regions, successfully reproduced the corresponding experimental data^12,18,62–68^. This comparison also suggests that the calculation by other codes is generally accurate except for Waligorski’s formula, which underestimates the dose by one order of magnitude at most. Around 100 nm of radius, LIon Track andChunxiang’s calculated data are higher than the others. Experimental data is not available at 100 nm but they overestimate the dose already at 49 nm. Therefore, the codes including ITSART are probably more accurate around 100 nm. That is, the radial distribution of energy deposited by 10 MeV protons (see Fig. 5b) indicates the validity of our model at high energies. No experimental data are available at this energy but the radial distribution calculated by our model is consistent with other calculation data. As the general trend, the radial distributions are characterised by a hump within 10 nm. This shape can be explained by the energy dependence of the electron range. The range of electrons increases with increasing electron kinetic energy. However, for energy ranging from 3 to 300 eV, the range (20 nm) is barely dependent on the energy^69^ and electrons are ejected forward with respect to the projectile. Therefore, the energy deposition is concentrated in a region within a radius of 20 nm, which is often called a track core.

**Figure 6:**
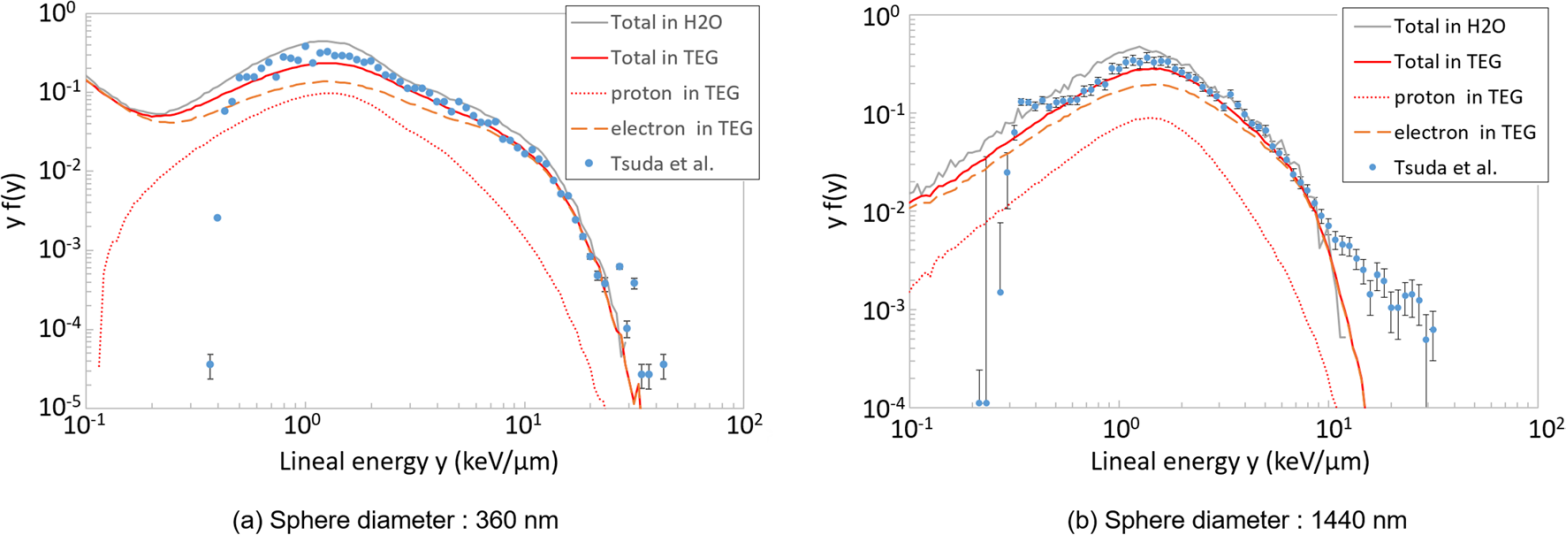
Measured and calculated lineal energy distribution of 30 MeV protons in TEG^71^.

The above comparisons (except for Fig. 3a) showed that, similar to other track-structure codes, our model can simulate the track structure of protons in liquid water. To confirm the advantage of our model, its performance in TEG was verified. Figure 6a and b show the lineal energy distribution of 30 MeV protons in 1.0 g/cm$^{3}$ TEG split into spheres with a diameters 360 nm and 1440 nm, respectively. In the experiment, the lineal energy was integrated by impact parameter from 0 to 1800 nm to represent that integrated over infinite impact parameter range. Note that *y* and *f*(*y*) denote the lineal energy and the probability density of *y*, respectively. As previously reported^71^, the sharp decrease in measured $y\times f$(*y*) at the lower ends is an artefact resulting from the detection lower limit and therefore the discrepancies below this limit are meaningless. The dotted and dashed curves show the fractions of $y\times f$(*y*) attributed to protons and electrons, respectively. Theoretically, the maximum lineal energy of the primary particle is equal to the linear energy transfer multiplied by 1.5 (this product is 2.9 keV/$\upmu$m for 30 MeV protons). Therefore, secondary electrons must be considered in explanations of the lineal energy distribution beyond 2.9 keV/$\upmu$m. The contribution of protons is noticeably lower than that of electrons. For example, focusing on $y=10$ keV/$\upmu$m, the lineal energy directly attributed to protons is no more than 2.9 keV/$\upmu$m and the rest ($\ge$ 7.1 keV/$\upmu$m) comes from electrons. In fact, Fig. 6a indicates that only $2\times 10^{-3}$ of ${\hbox{y}}\times {\hbox{f(y)}}$ out of the total $2\times 10^{-2}$ comes from protons at 10 keV/$\upmu$m.

**Table 1:** $y_{D}$ calculated from y distributions plotted in Fig. 6.

	360 nm	1440 nm	1440 nm ($\le \,10$ keV/$\upmu$m)
Experiment	7.49	7.43	4.07
ITSART(TEG)	7.56	4.28	4.09
ITSART(${{\hbox{H}}}_{2}{\hbox{O}}$)	7.17	3.95	3.88

**Figure 7:**
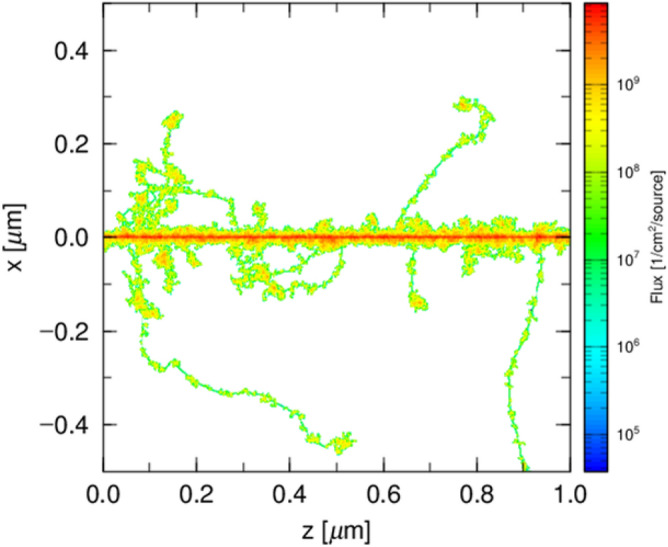
Track structure of 200 protons with initial energy of 30 MeV.

These tendencies indicate that the good agreement between the experiment and the calculation results from the accurate treatment of energetic secondary electron production events. Particularly, high lineal energy events are caused by energetic secondary electrons produced in an upstream sphere depositing its energy in the downstream spheres. In Fig. 6b, ${\hbox{y}}\times {\hbox{f(y)}}$ decreased above 3 keV/$\upmu$m because the range of electrons is shorter than that required for penetrating the 1440-$\upmu$m-diameter spheres and transferring the energy of secondary electrons to neighbouring cells. In other words, the energy lost by proton is deposited generally within the sphere.

To backup above analysis, the track structures of 200 protons starting at 30 MeV are shown in Fig. 7. As discussed elsewhere^72^, the secondary electron buildup occurs in the first 10 nm, which is negligibly thin compared to the size of the spheres. Several prominent secondary electron emissions are seen but the majority of secondary electrons remains in 30 nm as discussed concerning Fig. 5. These facts show that low energy secondary electrons are in equilibrium in the scale of 30 nm but the energetic secondary electrons are not even in the scale of $1\,\upmu$m. These facts well explain the presence of high y component in Fig. 6a which represents the probabilistic emission of energetic secondary electrons. The triangular ${\hbox{y}}\times {\hbox{f(y)}}$ spectrum in Fig. 6b represents charged particle balance being equilibrated in the scale of 1440 nm.

In Fig. 6a, the peak of the lineal energy occurred at approximately 1.3 keV/$\upmu$m whereas the stopping power of the 30 MeV proton calculated by ATIMA was 1.9 keV/$\upmu$m. This comparison indicates that the energy corresponding to 0.6 keV/$\upmu$m was transferred to neighbouring cells by secondary electrons. The rest was deposited directly by protons or deposited by secondary electrons with range short enough to be stopped in the same sphere. The difference of 0.6 keV/$\upmu$m, fairly reproduced by our model, symbolically shows the effects of secondary electrons on the lineal energy. To compare y distributions, one of the best indices is $y_{D}$. Table 1 shows $y_{D}$ of y distributions plotted in Fig. 6. As explained by Tsuda et al.^71,73^, y above 10 keV/$\upmu$m in Fig. 6b is an artefact attributed to structural materials such as the cathode wire. Therefore $y_{D}$ was calculated by integrating ${\hbox{y}}\times {\hbox{f(y)}}$ below 10 keV/$\upmu$m to exclude the contribution from the artefact. This suggests that $y_{D}$ calculated by ITSART assuming TEG target agrees with experiment within 1%. It is also illustrated that the consideration for the actual target composition is important since $y_{D}$ calculated assuming water target is systematically underestimated in both cases.

As explained in the “Methods2” section, compared with the conventional track-structure models based on dielectric functions, our model lacks explicit event-by-event treatment of non-ionising reactions. However, the above benchmark results illustrated that this has no effect on the important quantities required for micro-dosimetric calculations. The secondary electron energy spectra are irrelevant to non-ionisation events. The range calculation depends on the non-ionising energy loss. Nevertheless, the particle range calculated by ATIMA modulated to avoid double counting of ionisation energy loss agrees with ATIMA because the effects of the modulation are cancelled out along the trajectory. The radial dose distribution is also insensitive to non-ionising reactions because these reactions occur only along the track core. Explicit treatment of non-ionising reactions may affect lineal energy calculations but non-ionising reactions occurred extremely frequently such that a diameter of 360 nm (see Fig. 6a) was too long to view the discrete nature of these reactions. Based on the above verification, the accuracy and the versatility of ITSART were illustrated.

## Conclusion

In this study, a track-structure model applicable to any material was developed on the basis of stopping power systematics and a secondary electron energy distribution systematic formula. Despite the perturbation induced by stochastic secondary electron production, the calculated stopping range in liquid water agreed well with literature data, owing to the use of a restricted stopping power calculation algorithm. The calculated radial dose distribution in liquid water also agreed with the literature data indicating the accuracy of the secondary electron double-differential distribution and secondary electron transport by ETS-mode in downstream. A good agreement between the calculated lineal energy spectrum and the measurement data was realised by applying this model to the energy deposition of protons in TEG. This fact illustrates the validity of our model for materials other than liquid water.

Our model can perform track-structure calculations for arbitrary materials without knowledge of the dielectric function associated with the target material. These results revealed that the drawback of this approach, i.e. the implicit treatment of non-ionisation loss, has no effect on the above calculations. This versatile track-structure model is useful for calculating the microscopic energy deposition for materials other than liquid water. This model is useful for various domains (such as soft-error analysis, radiation biology, radiation therapy, and detector physics) of research. ITSART, the track-structure calculation method developed in this study, has been extended for heavy ions by scaling the cross section taking into account for the effective charge of the projectile and implemented into PHITS Version 3.25 and later.
